# ΔSUVmax adds prognostic value to early response assessment during the first-line treatment of classical Hodgkin lymphoma: a retrospective cohort study

**DOI:** 10.1186/s40644-025-00904-x

**Published:** 2025-07-01

**Authors:** László Imre Pinczés, Dávid Tóthfalusi, Boglárka Dobó, Sándor Barna, Bence Farkas, Ildikó Garai, Árpád Illés, Zsófia Miltényi

**Affiliations:** 1 Division of Hematology, Department of Internal Medicine, Faculty of Medicine, University of Debrecen, Debrecen, Hungary; 2 Doctoral School of Clinical Medicine, University of Debrecen, Debrecen, Hungary; 3 Division of Nuclear Medicine and Translational Imaging, Department of Medical Imaging, Faculty of Medicine, University of Debrecen, Debrecen, Hungary; 4 Scanomed Ltd, Debrecen, Hungary

**Keywords:** Hodgkin lymphoma, ΔSUVmax, SUVmax, Deauville score, PET/CT

## Abstract

**Background:**

In classical Hodgkin lymphoma (HL), optimizing early risk stratification and response assessment are the cornerstones of therapy. The advanced interpretation of positron emission tomography - computed tomography (PET/CT) results can provide prognostic information beyond the Deauville score (DS). The aim of our study was to explore the prognostic value of the change in maximum standardized uptake value (ΔSUVmax) to predict disease progression during the first-line treatment of adult HL.

**Methods:**

All patients were treated with curative intent, standard therapy. PET/CT assessments were performed at baseline, interim and end-of-treatment timepoints. ΔSUVmax cut-off values were determined by the receiver operating characteristics (ROC) analysis. Overall- (OS) and progression-free survival (PFS) were determined as primary endpoints.

**Results:**

Baseline SUVmax did not differ in patients who progressed during or after first-line therapy compared to patients in remission. However, patients with progressive disease had a higher mean SUVmax and lower ΔSUVmax at interim analysis. The presence of a ΔSUVmax > 88% after 2 cycles of therapy was associated with longer PFS (*P* = 0.013 [HR, 5.21]), with a negative predictive value exceeding the DS. The combination of ΔSUVmax with DS further stratified PET-negative patients: the 5-year PFS of low-risk and high-risk patients were 92.1% and 79.1%, respectively (*P* = 0.047 [HR, 2.87]). The ΔSUVmax cut-off of 55% in patients with DS 3–5 revealed high-risk patients with significantly lower 5-year OS and PFS (*P* = 0.008 [HR, 13] and *P* < 0.001 [HR, 11.5], respectively).

**Conclusions:**

Altogether, ΔSUVmax is a promising standalone prognostic marker or combination partner of DS in the early risk stratification and response assessment of HL.

**Supplementary Information:**

The online version contains supplementary material available at 10.1186/s40644-025-00904-x.

## Background

Approximately 20–30% of classical Hodgkin lymphoma (HL) patients experience primary refractory disease or relapse (R/R) after first-line treatment [1, 2]. With the advent of novel targeted therapies, optimizing early risk stratification and response assessment are becoming increasingly important to guide therapy. To enable a broader application of risk-stratified treatment, it is essential to identify biomarkers associated with response to first-line treatment and the risk of relapse thereafter.

Achieving complete metabolic remission after 2 cycles and completion of first-line therapy is an important predictor of overall survival (OS) and progression-free survival (PFS) in HL. These interim and restaging time points are used to evaluate early treatment response and have been used in several recent trials to guide treatment decisions. Response evaluation is guided by ^18^F-fluorodeoxyglucose (FDG) positron emission tomography– computed tomography (PET/CT) scans and is standardized with the Lugano classification, based on the measurement of FDG uptake with a 5-point scale system, i.e., the Deauville score (DS) [3, 4]. Interim PET/CT proved to be the prognostic marker with the highest predictive value, irrespective of other risk factors included in the International Prognostic Score [5].

Quantitative and semi-quantitative PET/CT analysis can provide prognostic information beyond staging and DS alone. The standardized uptake value (SUV) objectively reflects the metabolic activity of the tumor tissue, and the change in the maximum SUV (ΔSUVmax) correlates with disease activity, thus reflecting the effect of therapy. However, studies that have prospectively or retrospectively investigated ΔSUVmax in the first-line setting are scarce. Here, we present the results regarding the prognostic value of ΔSUVmax to predict disease progression during first-line treatment of HL.

## Methods

### Patients and study design

We retrospectively analysed the demographic data and clinical features of HL patients treated at the University of Debrecen between January 2010 and October 2021. Patients were aged 18 years or older and had histologically confirmed diagnosis of HL. Participants must have received standard combined chemotherapy without dose reduction, and only patients whose SUVmax value was reported during baseline and interim PET/CT scans were eligible for this analysis. No exclusion criteria regarding bone marrow and other organ function or Eastern Cooperative Oncology Group (ECOG) performance status were determined.

### Treatment

Patients were treated according to the evidence and consensus-based practice guidelines of the Hungarian Society of Hematology and Transfusion [6]. The standard combined chemotherapy treatment consisted of doxorubicin, bleomycin, vinblastine, and dacarbazine (ABVD regimen). Patients with early stage and favourable prognosis received 2 cycles of ABVD followed by involved field radiotherapy (IFRT) or 4 cycles of ABVD. Early stage with unfavourable prognostic factors was treated with 4 cycles of ABVD followed by IFRT or 6 cycles of ABVD. Patients with advanced-stage disease were treated with 6 cycles of ABVD with IFRT in patients with localized residuum on the end-of-treatment imaging. The omission of bleomycin after 2 cycles of ABVD was allowed in patients achieving a DS of 1–2 at the time of interim PET/CT assessment (ABVD + AVD regimen). In patients with impaired cardiac function, an equivalent dose of epirubicin was administered instead of doxorubicin (EBVD or EBVD + EVD regimens).

After the marketing authorisation of brentuximab vedotin (BV), HL patients with a Lugano staging classification stage of IV may received BV given together with doxorubicin, vinblastine, and dacarbazine (A + AVD). The acquisition of brentuximab vedotin through an individual fairness request has made it necessary for some patients to start treatment according to the ABVD protocol. In such cases, the switch from bleomycin to brentuximab vedotin was only allowed after approval had been obtained (ABVD + A + AVD regimen).

### PET/CT scan

Analysis of the PET/CT scans was conducted according to the guidelines of the European Association of Nuclear Medicine (EANM) [7]. PET/CT assessments were performed at baseline, after 2 cycles of first-line therapy, and 6 to 8 weeks after completion of first-line treatment. Analysis of imaging data was performed by 3 nuclear medicine physicians (S.B., B.F., and I.G.). Lesions were identified by visual assessment, while SUVmax was determined by drawing a region of interest around the intense pathologic uptakes. SUVmax was defined as the most active voxel in each PET/CT scan, after manual delineation of tumor regions, regardless of its location. ΔSUVmax (%) was calculated with the following formula: (baseline SUVmax– interim SUVmax)/baseline SUVmax × 100. In patients without measurable metabolic activity during interim PET/CT, SUV was set to 0 and ΔSUVmax to 100%. Response assessment was performed according to Lugano classification and interpreted by the Deauville 5-point scoring system.

### Endpoints

OS was defined as time from interim PET/CT scan until death from any cause. PFS was calculated from the day of interim assessment to progressive disease or death as a result of any cause.

### Statistical analysis

Categorical variables are given as their frequencies and percentages, while continuous variables with medians and ranges. The Kolmogorov–Smirnov test was used for the evaluation of data normality. Continuous variables were evaluated using the Mann–Whitney U test or t-test based on the normality of the data. The receiver operating characteristics (ROC) analysis was used to identify the optimal cutoff value of ΔSUVmax for survival prediction. Simple linear regression was computed to assess the relationship between ΔSUVmax and survival endpoints. Survival functions were calculated by using Kaplan-Meier estimates, and the comparison between categories was made using the log-rank test. The level of statistical significance was considered at *p* < 0.05. Statistical analyses were performed using SPSS 26.0 (IBM Corp., Armonk, NY, USA).

### Ethics approval and consent to participate

The study protocol followed the ethical guidelines of the 1975 Declaration of Helsinki, and ethical approval was obtained from the institutional review board of University of Debrecen, Debrecen, Hungary (IRB number: 4881). Written informed consents were obtained from all patients prior to treatment.

## Results

### Patients and follow-up

Between August 2010 and October 2023, 118 newly diagnosed HL patients had sufficient baseline, imaging, and follow-up data for analysis. All patients were suitable for ΔSUVmax calculation. The baseline characteristics of the population are described in Table 1. With a median follow-up of 6.1 years, there were 10 OS and 25 PFS events. The 5-year OS and PFS of the whole study group were 92.5% and 80%, respectively.

**Table 1:** **Patient characteristics** *ABVD– doxorubicin*,* bleomycin*,* vinblastin*,* dacarbazine*,* EBVD– epirubicin*,* bleomycin*,* vinblastin*,* dacarbazine*,* A + AVD– brentuximab vedotin*,* doxorubicin*,* vinblastin*,* dacarbazine*,* EORTC - European organisation for research and treatment of Cancer*,* GHSG– German hodgkin study Group*,* SUV– standard uptake value*,* SUVmax– maximal standard uptake value*

Characteristics		Data
Age, median, years		34 (18–75)
Male / female, n		60 / 58
Histological subtype, n (%)		
	Nodular sclerosis	66 (56)
	Mixed cellularity	31 (26)
	Lymphocyte rich	11 (9)
	Lymphocyte depleted	0
	Not determined	10 (9)
Ann Arbor stage, n (%)		
	I	4 (3)
	II	38 (32)
	III	15 (13)
	IV	61 (52)
EORTC stage, n (%)		
	Early favorable	10 (8)
	Early unfavorable	32 (27)
	Advanced	76 (65)
GHSG stage, n (%)		
	Early	13 (11)
	Intermedier	23 (19)
	Advanced	82 (70)
B-symptoms, n (%)		70 (59)
Extranodal involvement, n (%)		52 (44)
Spleen involvement, n (%)		32 (27)
Mediastinal bulky, n (%)		35 (30)
First-line treatment, n (%)		
	ABVD	98 (83)
	ABVD + AVD	3 (2)
	EBVD	5 (4)
	EBVD + EVD	1 (1)
	A + AVD	9 (8)
	ABVD + A + AVD	2 (2)
Staging SUVmax, mean, n (range)		12,83 (4,4–26)
Interim SUVmax, mean, n (range)		2,14 (0–10,1)
ΔSUVmax, mean, % (range)		82,6 (29,5-100)
Interim Deauville score, n (%)		
	1	36 (30)
	2	48 (41)
	3	24 (20)
	4	8 (7)
	5	2 (2)

### SUVmax analysis

**Fig. 1:**
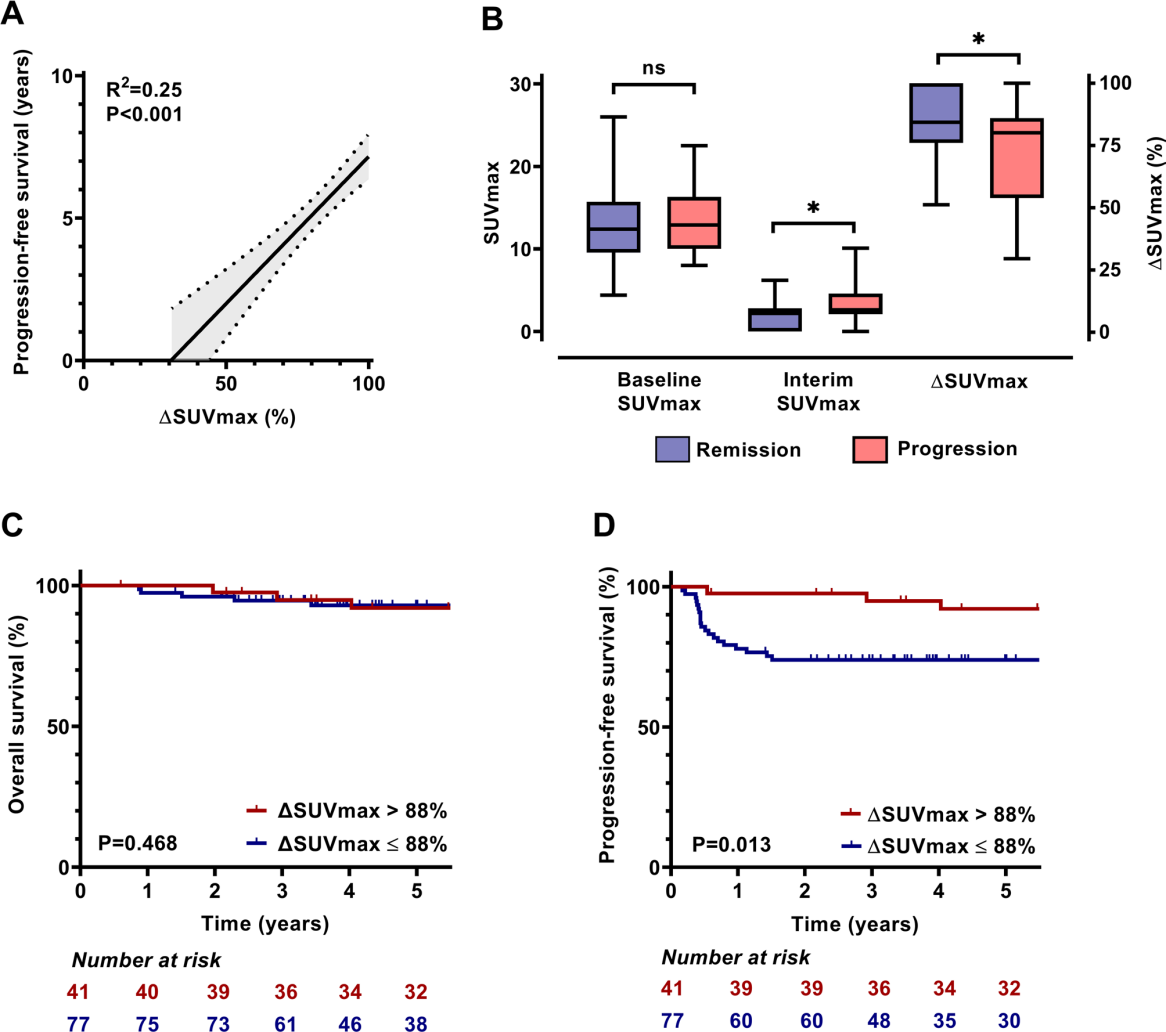
Correlation, evolution, and impact of SUVmax parameters on outcome. **(A)** The regression line and 95% confidence band around the line for the mean value of ΔSUVmax and progression-free survival. **(B)** SUVmax on the PET/CT at baseline and interim measurement, and ΔSUVmax regarding baseline and interim PET/CT, stratified for patients in remission after 5-years of follow-up compared to patients with progressive disease during or after treatment. **C-D.** Kaplan-Meier overall survival and progression-free survival analysis for interim ΔSUVmax cutoff at 88%. *SUV– standard uptake value*,* SUVmax– maximal standard uptake value*, * - *p* < 0.001,* ns– not significant*

The increase in ΔSUVmax significantly predicted PFS (R^2^ = 0.25, *p* < 0.001) (Fig. 1A), and OS (R^2^ = 0.12, *p* < 0.001). Baseline SUVmax did not differ in patients who progressed during or after first-line therapy compared to patients in remission (13.27 vs. 12.71, *p* = 0.572). However, progressive patients showed a higher mean SUVmax (3.43 vs. 1.80, *p* < 0.001) and lower ΔSUVmax (72.1% vs. 85.4%, *p* < 0.001) after 2 cycles of the first-line regimen (Fig. 1B).

From the results of ROC analysis, the best ΔSUVmax cutoff was 88%, regarding the whole population for OS and PFS. The presence of high ΔSUVmax (> 88%) was associated with longer PFS, but not with OS. The 41 patients with a high ΔSUVmax had a significantly better outcome with a 5-year PFS of 92.1% vs. 74% for patients with a lower ΔSUVmax (*p* = 0.013 [hazard ratio; HR, 5.21])(Fig. 1C-D). The sensitivity and specificity of the ΔSUVmax cutoff were 84% and 39.8% for PFS, respectively, with a positive predictive value (PPV) of 27.3% and a negative predictive value (NPV) of 90.2% (Table 2). Further stratification of patients to early stages and advanced stage disease revealed no difference in OS, however it is notable that no OS events happened in early stage patients with ΔSUVmax > 88% (Suppl. Figure 1). The PFS benefit observed in the overall population was also confirmed in patients with advanced stage disease. Regarding baseline SUVmax, no significant cutoff value for survival prediction could be identified.

**Table 2:** Performance of PET/CT assessment parameters

	Sensitivity (%)	Specificity (%)	PPV (%)	NPV (%)
**Deauville score**	24.0	95.7	60.0	82.4
**ΔSUVmax 88%**	84.0	39.8	27.3	90.2
**Combined score**	24.0	98.9	85.7	82.9

Performance of prognostic models for PFS at the interim assessment of PET/CT. The combined score stands for the combination of a Deauville score of 1–2 vs. Deauville scores of 3–5, separated by a ΔSUVmax cutoff of 55%. *PPV– positive predictive value*,* NPV– negative predictive value*,* SUVmax– maximal standard uptake value*,* PET/CT– positron emission tomography with computed tomography*,* PFS– progression-free survival*

### Combination of the Deauville score and ΔSUVmax

**Fig. 2:**
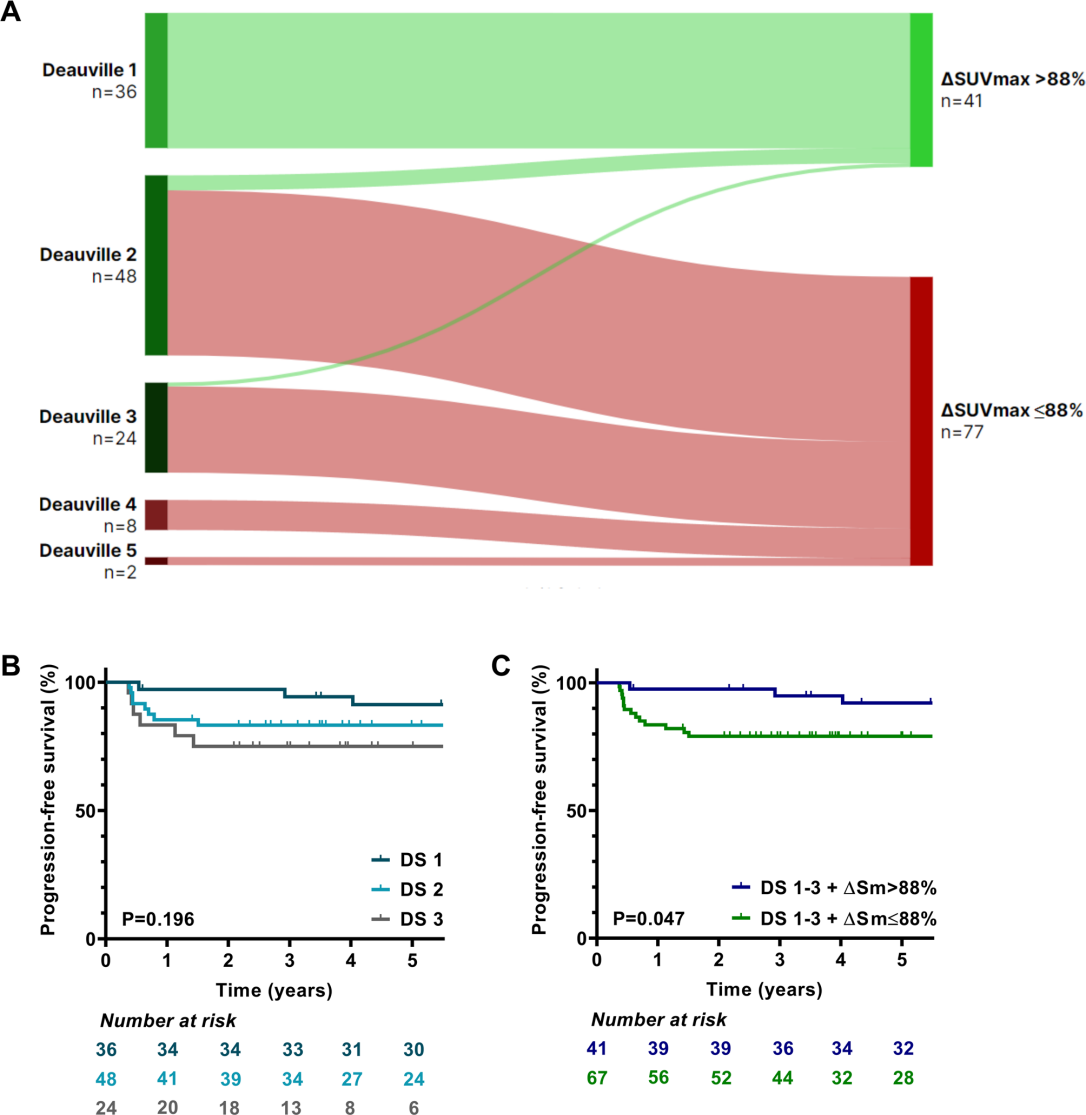
Baseline and interim PET/CT parameters. **A.** Sankey plot of subgroup classification based on interim PET/CT results regarding the Deauville 5-point scale and ΔSUVmax cutoff at 88%. **B-C.** Kaplan-Meier progression-free survival analysis for the Deauville 5-point scale, regarding negative interim PET/CT results, and the interim ΔSUVmax cutoff at 88% *SUVmax– maximal standard uptake value*

Interim PET/CT scan reported with the Deauville criteria was positive (DS of 4–5) in 9% of the cases. Positive interim PET/CT results were predictive of inferior OS and PFS (*p* = 0.003 [HR, 8.16] and *p* < 0.001 [HR, 5.48], respectively, data not shown). Patients with positive and negative interim PET/CT results had a 5-year OS of 68.6% vs. 94.8%, and a 5-year PFS of 40% vs. 83.7%, respectively. The outcomes of patients with a DS of 1, 2, and 3 did not differ from each other (Fig. 2B).

The presence of low ΔSUVmax reclassified more than 60% of patients with a DS of 1–3 to a high-risk group (Fig. 2A). Regrouping patients with DS of 1–3, according to the 88% cutoff of ΔSUVmax resulted in a significant association with PFS, but not with OS (Fig. 2C). The 5-year PFS of low-risk and high-risk patients with negative interim PET/CT scans were 92.1% and 79.1%, respectively (*p* = 0.047 [HR, 2.87]).

**Fig. 3:**
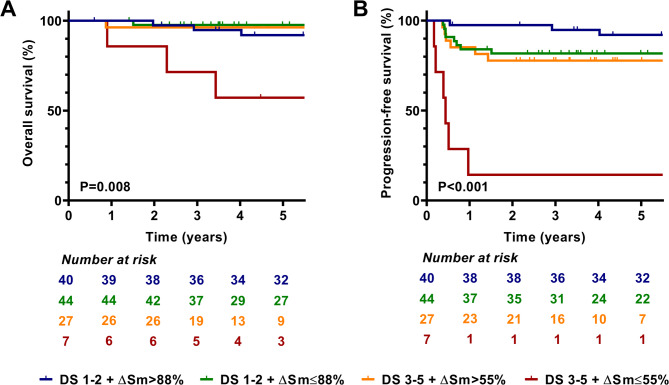
Modified approach to the deauville score, combined with ΔSUVmax parameters. Kaplan-Meier overall- **(A)** and progression-free **(B)** survival analysis for the Deauville 5-point scale, with a cutoff between Deauville 1–2 and Deauville 3–5 scores, combined with 88% and 55% ΔSUVmax cutoffs, respectively *SUVmax– maximal standard uptake value*

The incorporation of patients with an interim PET/CT with a DS of 3 to the positive (DS of 4–5) patient population enabled different ROC analysis for patients with a DS of 3–5. The best ΔSUVmax cutoff was 55% for OS and PFS, regarding this subgroup (*p* = 0.008 [HR, 13] and *p* < 0.001 [HR, 11.5], respectively) (Fig. 3). Patients who had both an interim PET/CT scan with a DS of 3–5 plus a ΔSUVmax lower than 55% showed significantly lower 5-year OS and PFS compared to patients with DS 1–2 (57.1% vs. 94.7%, *p* < 0.001 [HR, 12.7] and 14.3% vs. 86.4%, *p* < 0.001 [HR, 12.6], respectively) and with DS 3–5 plus ΔSUVmax > 55% (57.1% vs. 96.3%, *p* = 0.009 [HR, 13.1] and 14.3% vs. 77.8%, *p* < 0.001 [HR, 7.4], respectively). The interim status of DS 1–2 and DS 3–5 plus ΔSUVmax > 55% did not significantly discriminate patients. Also, the previously defined ΔSUVmax cutoff of 88% did not separate patients with a DS of 1–2.

No other significant individual or combined cutoff values for survival prediction could be identified regarding DS and ΔSUVmax subgroups.

## Discussion

It is well known that the neoplastic component of HL is situated within an abundant mixture of non-neoplastic inflammatory cells, with malignant Reed-Sternberg cells comprising less than 1–2%. Therefore, PET/CT proved to be valuable in assessing tumor burden by examining the functionally active volume of the tumor, as opposed to merely recognizing the entire visible mass. Quantification of the activity of the infiltrative component is crucial because changes in volumetric parameters representing tumor volume and tumor burden can indicate treatment response and serve as a surrogate marker for outcome. Because of its predictive value, interim PET/CT has been used in several recent trials in early- and advanced-stage HL [8, 9]. However, a small proportion of treatment failure has been observed even in patients with negative PET/CT scans and good baseline prognostic values, which underlines the importance of further refinement of the response criteria.

The widely accepted objective tool for evaluating PET/CT is the DS. The DS is calculated by visually comparing the FDG uptake (via SUV) in tumor areas with that in the liver and mediastinal blood pool. However, visual assessment of DS often leads to inter-observer disagreement. In this regard, several attempts have been made to develop quantitative and semi-quantitative PET/CT grading methods that can improve the prognostic value of DS [10, 11]. These include the ΔSUVmax, which measures the efficacy of therapy through a decrease in FDG uptake and is able to identify subsets of patients with significantly different outcomes [12].

Metabolic tumor volume (MTV) has also been identified as an important prognostic factor, particularly in the first-line setting for HL [13]. Although MTV is a much more comprehensive clinical parameter and provides a deeper understanding of disease progression than ΔSUVmax, its everyday use is still fraught with technical obstacles. In most studies, a different cutoff for MTV is used without validation of results, and several calculation methodologies are applied by the available semi-automatic analysing software. Furthermore, the possibility of obtaining software and the expertise required to use it is not harmonised between smaller and larger centers. These factors impede the measurement of MTV in everyday practice and the use of this parameter as a prognostic marker. Meanwhile, the SUVmax value is part of the reporting of PET/CT scans performed during routine assessment. Its change during the course of the disease can be calculated on the spot with a simple formula and does not require the involvement of a nuclear medicine specialist.

This study clearly shows the prognostic value of ΔSUVmax in patients with HL, homogenously treated with a risk-adapted treatment strategy. As a standalone prognostic marker, ΔSUVmax is a good surrogate for progression-free survival by effectively separating patients with lower and higher risk of disease progression. Combined with the Deauville 5-point scale, ΔSUVmax can further stratify PET-negative and PET-positive patients at the time of interim measurement. Low-risk patients were identified by a high ΔSUVmax and a negative interim PET/CT scan, as ΔSUVmax was able to discriminate high-risk patients within the PET-negative population determined by the DS. This observation emphasizes the additional value of ΔSUVmax to visual analysis. Further evaluation with different treatment approaches and PET/CT methodology is also worth considering [14, 15].

Since, unlike the parametric ordinal DS, ΔSUVmax is a dynamic variable, deviating from the categorical classification in terms of positivity and negativity regarding DS can be an exciting approach worth considering. Compared with a lower threshold, the cutoff between DS 3 and 4 has a higher prognostic accuracy and at the same time, a lower inter-observer variation. However, patients with a DS of 3 are still the most vulnerable to a lack of inter-observer agreement, and thus to type II error during assessment. Incorporation of patients with a DS of 3 into the PET-positive population and separating them by a predictive ΔSUVmax cutoff of 55% identified high-risk patients for low OS and PFS. These patients could be those who benefit from early escalation of first-line therapy.

The ΔSUVmax values found in this study are in accordance with data already available [16–19]. Nevertheless, we must note that ΔSUVmax (similarly to MTV) acts as a continuous nonlinear variable, and as such, various cutoff values have been suggested to predict treatment outcomes. Rossi and colleagues reported a cutoff value of ΔSUVmax for PFS prediction of 71%, with superior sensitivity (54% vs. 46%) and specificity (94% vs. 84%) compared to DS [16]. More recently, Yang et al. reported a superior sensitivity (85% vs. 62%) and comparable specificity (82% vs. 88%) to DS with the cutoff value of 83% [17]. Discrimination at 93% resulted in balanced sensitivity (70%) and specificity (77%) as opposed to DS (10% and 98%, respectively) for Riberio and colleagues [18]. In a pediatric population of HL patients, Ibrahim et al. found similar sensitivity (83% vs. 80%) and inferior specificity (83% vs. 100%) with a ΔSUVmax cutoff of 56.3%, compared to DS [19]. As previous reports and our own experience have demonstrated, tailoring the cutoff of ΔSUVmax and determining the appropriate combination with Deauville scores can help improve the predictive value of the Deauville 5-point scale. Additional studies are needed to confirm these optimal cutoffs for different clinical scenarios in HL before ΔSUVmax can be used for clinical decision-making.

We acknowledge the limitations of this non-randomized study, such as the small sample size and low number of events. Favorable OS may be partially explained by the use of high-dose chemotherapy followed by autologous hematopoietic stem cell transplantation and novel therapies in patients who failed first-line therapy. Also, in our opinion, real-world data is of particular importance in this setting. Our results need to be evaluated on an independent cohort prior to generalized adaptation of the results.

In conclusion, our data suggest that interim PET/CT response assessed by the Deauville 5-point scale should be discussed in the light of ΔSUVmax values. With the advent of highly effective novel therapies, one of the next goals for clinical trials is to investigate whether some HL patients can possibly be cured with reduced or omitted doses of classical chemotherapeutic agents. ΔSUVmax can be a crucial element of PET-adapted prospective studies aiming to identify adequate candidates for this approach. Also, determining ΔSUVmax is an accessible and reproducible marker in everyday practice that can draw attention to patients requiring more rigorous monitoring.

## Electronic supplementary material

Below is the link to the electronic supplementary material.

Supplementary Material 1

## Data Availability

The datasets used and analysed during the current study are available from the corresponding author on reasonable request.

## Abbreviations

ΔSUVmax: Change in maximum standardized uptake value
A + AVD: Doxorubicin, brentuximab vedotin, vinblastine, and dacarbazine
ABVD: Doxorubicin, bleomycin, vinblastine, and dacarbazine
AVD: Doxorubicin, vinblastine, and dacarbazine
BV: Brentuximab vedotin
DS: Deauville Score
EANM: European Association of Nuclear Medicine
EBVD: Epirubicin, bleomycin, vinblastine, and dacarbazine
ECOG: Eastern Cooperative Oncology Group
EVD: Epirubicin, vinblastine, and dacarbazine
FDG: ^18^F-fluorodeoxyglucose
HL: Classical Hodgkin lymphoma
IFRT: Involved field radiotherapy
MTV: Metabolic tumor volume
NPV: Negative predictive value
OS: Overall survival
PET/CT: Positron emission tomography / computed tomography
PFS: Progression-free survival
PPV: Positive predictive value
R/R: Primary refractory or relapsed disease
ROC: Receiver operating characteristics
SUV: Standardized uptake value
