# Supplementary material for: ΔSUVmax adds prognostic value to early response assessment during the first-line treatment of classical Hodgkin lymphoma: a retrospective cohort study
Source: Cancer Imaging. 2025 Jul 1;25:80. doi: 10.1186/s40644-025-00904-x (PMC12210809; doi:10.1186/s40644-025-00904-x)
Supplement: Supplementary file 1 — Supplementary Material 1 [file 40644_2025_904_MOESM1_ESM.docx]

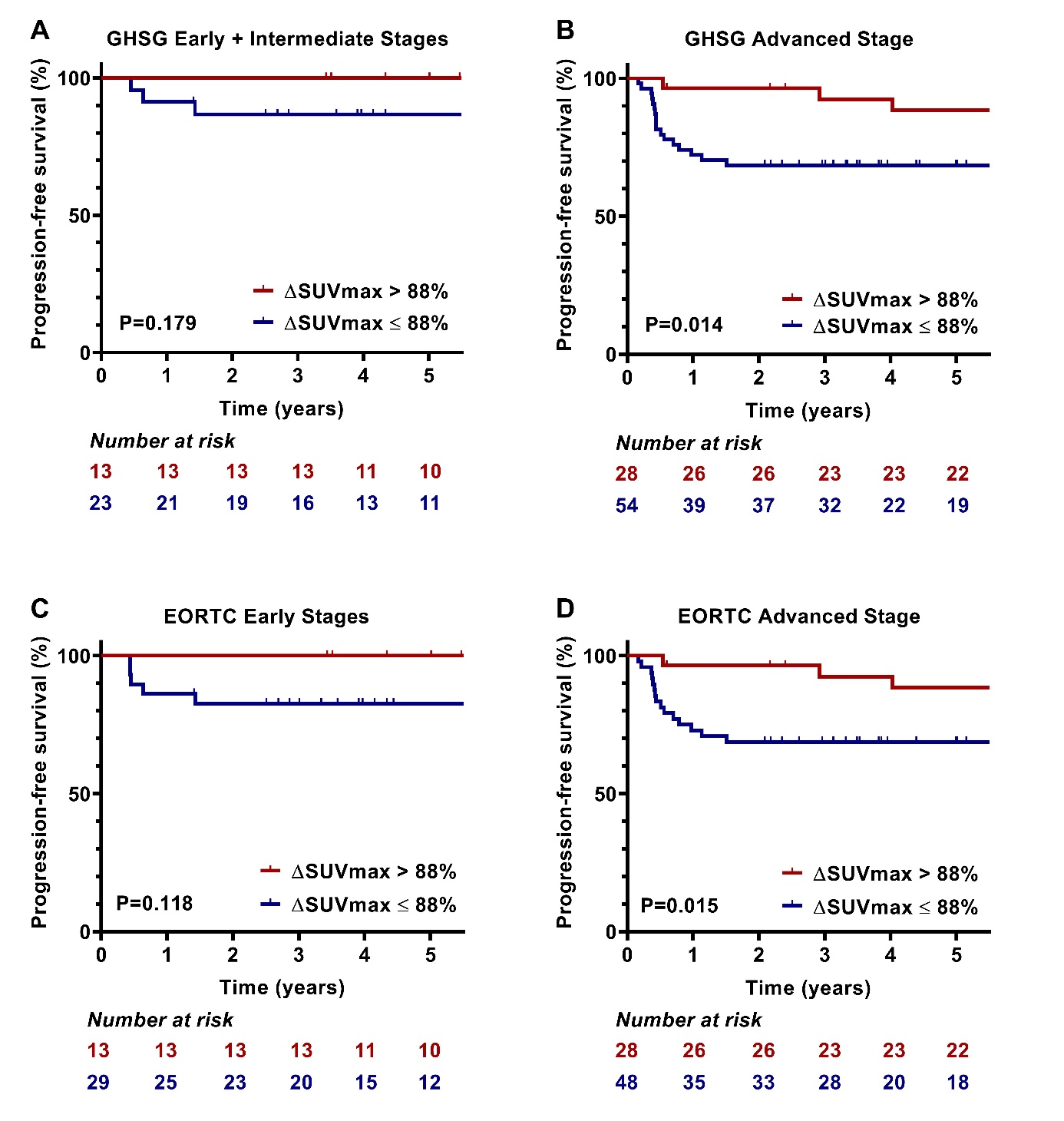


**Supplementary Figure 1. Impact of SUVmax parameters on outcome in early stages and advanced stage disease**

Kaplan-Meier overall survival and progression-free survival analysis for interim ∆SUVmax cutoff at 88% in different disease stages according to the German Hodgkin Study Group (GHSG) (A-B) and the European Organisation for Research and Treatment of Cancer (EORTC).

*EORTC - European Organisation for Research and Treatment of Cancer, GHSG – German Hodgkin Study Group, SUVmax – maximal standard uptake value*
